# Case 2/2018 - 73-Year-Old Male with Ischemic Cardiomyopathy, Cachexia and Shock

**DOI:** 10.5935/abc.20180065

**Published:** 2018-04

**Authors:** Rafael Amorim Belo Nunes, Jussara de Almeida Bruno, Hilda Sara Monteiro Ramirez, Léa Maria Macruz Ferreira Demarchi

**Affiliations:** Instituto do Coração (InCor) do Hospital das Clínicas da Faculdade de Medicina da Universidade de São Paulo (HC-FMUSP), São Paulo, SP - Brazil

**Keywords:** Atherosclerosis, Heart Failure/physiopathology, Cardiomyopathy, Dilated/complications, Weight Loss, Cachexia

The patient is a 73-year-old male, born in the municipality of Jacupiranga, SP, and coming from São Paulo city, SP, complaining of 30-kg weight loss in the previous 4 months and worsening of his general state of health in the previous 24 hours.

He reported having coronary artery disease, with two episodes of infarction and one coronary angioplasty with stent implantation 8 years before. He had been diagnosed with ischemic cardiomyopathy and ejection fraction of 22%.

He was using spironolactone, losartan, carvedilol, furosemide and propatylnitrate.

His physical examination (April 29, 2004) showed emaciation, dehydration, heart rate of 80 bpm, inaudible blood pressure, increased jugular venous pressure, lungs with inspiratory wheezes, regular heart rhythm and no heart murmur on cardiac auscultation, liver palpable 3 cm from the right costal margin, and mild edema of the lower limbs. The patient received 1500 mL of 0.9% saline solution, which increased his blood pressure to 90/70 mm Hg.

The results of his laboratory tests (April 30, 2004) were as follows: hemoglobin, 17.2 g/dL; platelets, 99000/mm^3^; leukocytes, 7850/mm^3^; urea, 122 mg/dL; creatinine, 2.2 mg/dL; potassium, 6.5 mEq/L; sodium, 143 mE/L. His arterial blood gas analysis was as follows: pH, 7.3; bicarbonate, 16 mEq/L; and base excess, (-)7 mEq/L.

His electrocardiogram (April 29, 2004) (Figure 1) showed sinus rhythm, heart rate of 68 bpm, PR of 200 ms, dQRS of 120 ms, QT of 440 ms, left atrial overload and indirect signs of right overload (Peñaloza-Tranchesi), in addition to left anterior hemiblock. No pathological Q wave was seen.

**Figure 1:**
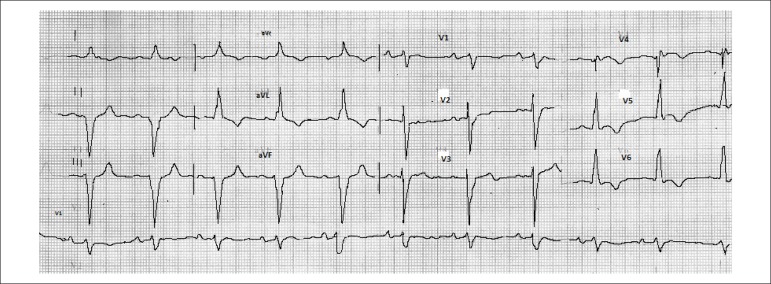
ECG: left atrial overload and indirect signs of right atrial overload (Peñaloza-Tranchesi), in addition to left bundle-branch block and left anterior hemiblock.

He was admitted to the Hospital Auxiliar de Cotoxó to compensate his heart failure and acute renal failure.

The patient progressed with oliguria, dyspnea, and, on the third day of admission, he had sudden lowering of consciousness, fever and respiratory failure, requiring endotracheal intubation.

His previous laboratory tests on that same day were as follows: hemoglobin, 14.5 g/dL; leukocytes, 8500/mm^3^; sodium, 139 mEq/L; potassium, 3.7 mEq/L; urea, 170 mg/dL; creatinine, 2.2 mg/dL; leukocyturia, 10000/mL; and hematuria, 280000/mL.

During that episode, the findings were as follows: heart rate, 75 bpm; blood pressure, 100/60 mm Hg; temperature, 37.8ºC; arterial saturation, 97%; and crepitant rales at pulmonary bases. His heart rhythm was regular, with neither murmur nor accessory heart sound. His capillary glycemia was 166 mg/dL.

The patient was referred to the emergency unit of Incor. Pulmonary aspiration and stroke were his clinical suspicions.

His physical examination on admission (April 3, 2004) revealed an agitated and intubated patient, with heart rate of 90 bpm, blood pressure of 68/49 mm Hg, respiratory rate of 36 bpm, lungs with diffuse rhonchi, no abnormality on cardiac auscultation. His liver was palpated 3 cm from the right costal margin. There was edema (+++) of the lower limbs, with no signs of calf swelling.

Sedation was prescribed, as were dobutamine, noradrenaline, enoxaparin, vancomycin and imipenem/cilastatin.

His cranial tomography (May 4, 2004) showed a right occipital low attenuation area, widening of the cortical sulci, and no other significant change, findings compatible with right occipital ischemic stroke.

The patient remained shocked despite the administration of vasoactive amines, had bradycardia and asystole, and died (May 5, 2004; 16 h).

## Clinical aspects

The patient here reported is a male elderly with ischemic cardiomyopathy, and significant weight loss in the previous 4 months, in addition to worsening of his general state of health and clinical instability in the 24 hours prior to admission. Some diagnostic possibilities could explain his significant weight loss.

Cardiac cachexia is a frequent complication in the advanced stages of congestive heart failure (CHF) and associates with shorter survival.^1^ The physiopathology of that disorder has been first described by Pitman and Cohen^2^ Later, new evidence showed that the cause is multifactorial, related mainly to anorexia, a change in the routine of food uptake, in absorption and in the metabolism of patients with heart failure.^3^ Up to 34% of the patients with CHF being followed up on an outpatient basis are estimated to develop cardiac cachexia in the medium to long run.^4^ The deleterious effects of that condition impair the cardiac and respiratory functions and decrease immunity, which leads to higher mortality. Our patient’s systemic complications that hindered the reversion of his final findings might have been aggravated by cardiac cachexia. Regarding treatment, the literature reports many simultaneous actions that fight the most critical points in cachexia, such as nutritional therapy and the use of appetite stimulants, correction of anemia and edema, use of anabolic steroids and immunomodulation. Parallel to drug therapy, physical activity is indicated to maintain skeletal musculature,^5^ respecting the limits imposed by the disease and requiring strict follow-up.

Another possible explanation for cachexia would be the development of malignant neoplasia in a patient previously debilitated by heart disease. New studies have recently shown a higher interaction between oncologic and cardiac diseases, no longer attributed to diagnostic coincidence, but to an interaction between their morbidities. A recent publication^6^ has shown that the overlap between those two diseases results from the addition of shared risk factors, such as obesity, smoking, sedentary lifestyle and diabetes mellitus. In that context, the term cancer-related ‘cardiac cachexia’ appears. That complication is part of the natural history of neoplasms, leading to a progressive muscle mass loss (cachexia). However, many patients experience myocardial changes related to atrophy, remodeling and dysfunction, a set of findings known as cardiac cachexia.^7^

Insidious infectious diseases, such as tuberculosis and infectious endocarditis, can also be accompanied by consumptive findings. Usually, the manifestation most commonly associated with those conditions is fever (70% to 90% of the cases), which, in our patient, was only reported during hospitalization, but not in the last months of his disease. In addition, other symptoms and signs, such as cough, productive expectoration, nocturnal sweating or skin lesions (petechiae and subungual hemorrhages), lack.

On admission to the emergency service, the patient had signs of dehydration, arterial hypotension and systemic congestion, such as high jugular venous pressure, hepatomegaly and edema of the lower limbs. Such findings suggest a state of low cardiac output, which can correspond to an advanced stage of the underlying heart disease or decompensation associated with other contributing factors. On the admission electrocardiogram, signs of overload of the right chambers stand out, which might suggest a sudden increase in the pressures of the right atrium and ventricle, as observed in cases of acute pulmonary thromboembolism. Other factors that might contribute to acute decompensations in patients with chronic diseases and potentially immunosuppressed are bacterial infections, such as pneumonia and urinary tract infections.

Despite the treatment instituted, the patient’s clinical status worsened with renal failure and metabolic acidosis. On the third day of hospitalization, he had lowering of consciousness, fever, oliguria and respiratory failure, being submitted to endotracheal intubation. These findings initially suggest infectious decompensation and a possible toxic-metabolic process, but cranial tomography revealed a right occipital low attenuation lesion compatible with acute ischemic stroke. In patients with structural heart disease, ischemic encephalic injuries are commonly secondary to cerebrovascular atherosclerotic disease or episodes of cardioembolism in the presence of atrial fibrillation or other intracardiac thrombi. Less frequently, the cardioembolic phenomenon can be related to infectious endocarditis or cardiac tumors. (Rafael Amorim Belo Nunes, MD, Jussara de Almeida Bruno, MD, and Hilda Sara Monteiro Ramirez, MD)

**Diagnostic hypotheses:** ischemic cardiomyopathy, cardiac cachexia, acutely decompensated chronic heart failure (progression of the underlying disease? pulmonary thromboembolism? subjacent infection?), ischemic stroke (atherothrombosis? cardioembolic?). (Rafael Amorim Belo Nunes, MD, Jussara de Almeida Bruno, MD, and Hilda Sara Monteiro Ramirez, MD)

## Postmortem examination

**Figure 2:**
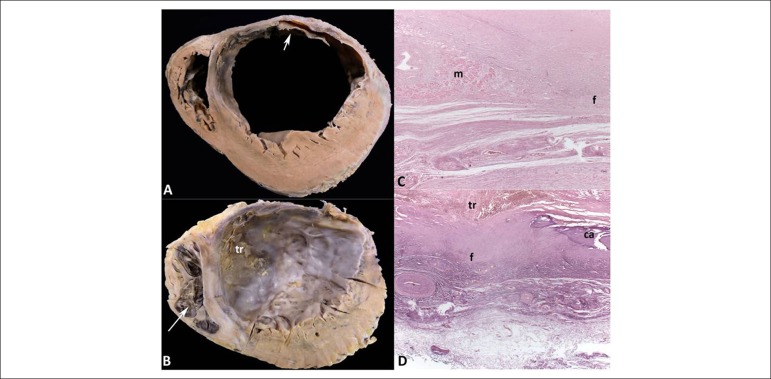
A and B: Cross sections of the ventricles in the middle-basal and apical regions, respectively. Healed transmural infarction in the left ventricular anterior and lateral walls and in the anterior two-thirds of the ventricular septum, with aneurysmal formation. Myocardial hypertrophy of the left ventricular walls not affected by the infarction is evident. Organizing thrombus in the endocardium of the left ventricular anterior and septal walls (arrow) in the middle-basal region, extending to the apical region (tr). Organizing thrombus in the right ventricular endocardium of the apex (arrow). C and D: Histological sections of the left ventricular aneurysm showing an isolated group of cardiomyocytes (m) and focal calcification (ca) amid mural fibrosis (f). Hematoxylin-eosin, 25x.

**Figure 3:**
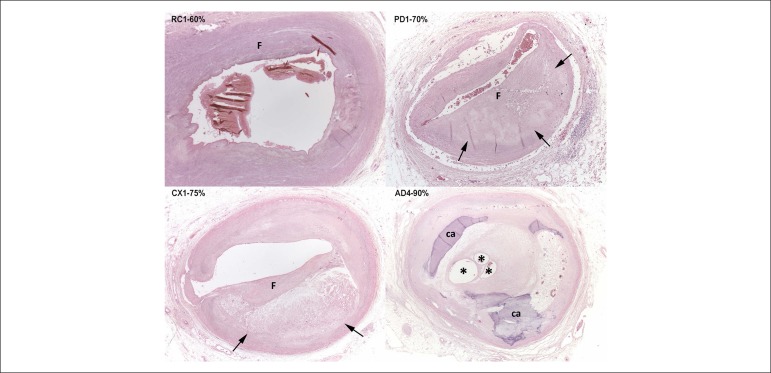
Histological sections of epicardial coronary arteries. Calcified atherosclerosis with luminal obstruction greater than 50% in the major branches. In the first centimeter of the right coronary artery (RC1), there is diffuse intimal fibrosis, with no lipid. In the first centimeter of the posterior interventricular branch of the right coronary artery (PD1) and of the circumflex branch (CX1), there are atherosclerotic plaques with fatty center and cholesterol crystals (arrows), surrounded by fibrosis (F). In the fourth centimeter of the anterior interventricular branch (AD4), there is luminal occlusion by an old recanalized thrombus, with multiple lumina and small vessels formed in the repairing process (*). Hematoxylin-eosin, 25x (RC1, CX1 and AD4) and 50x (PD1).

**Figure 4:**
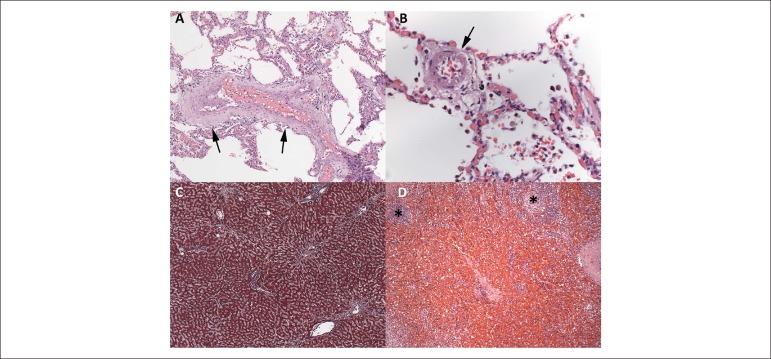
Chronic passive congestion. Lungs: thickening and tortuosity of the veins (A) and muscularization and hypertrophy of the media layer of an intra-acinar arteriole (B). Liver: sinusoidal dilatation in centrilobular areas (C). Spleen: intense congestion and widening of the red pulp; small, non-reactive lymphoid follicles (*). Hematoxylin‑eosin, 100x (A and D) and 400x (B). Masson trichrome, 50x (C).

**Figure 5:**
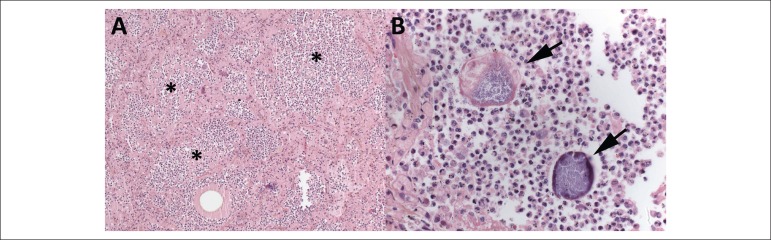
Lungs (A and B). Aspiration pneumonia: alveolar spaces filled with dense suppurative neutrophilic inflammatory infiltrate (*), amid which, particulate food material and filamentous bacterial aggregates, morphologically compatible with Actinomyces (arrows), can be seen. Hematoxylin-eosin, 100x (A).

The external examination revealed significant weight loss and moderate edema in the subcutaneous tissue, more marked in the lower limbs. On opening of the abdomen, 280 mL of yellow translucent ascitic fluid escaped. The heart weighed 644g (normal: 300-350g), and both ventricles were enlarged. Cross sections of the ventricles evidenced healed transmural myocardial infarction in the left ventricular anterior and anterolateral walls, involving at least 45% of the left ventricular myocardial mass and the anterior two-thirds of the ventricular septum, from the heart base to its tip (Figure 2). Significant left ventricular aneurysmatic dilatation was observed, with important fibrosis and thinning of the anterior wall, whose thickness ranged from 0.2 cm to 1.4 cm. In the middle and apical thirds of the left ventricle, an organizing laminated thrombus was identified, adhered to the endocardial surface of the anterior wall and ventricular septum (Figure 2). The myocardium not affected by the infarction in the left ventricle was hypertrophied. The right ventricle showed moderate mural hypertrophy and dilatation, with an organizing thrombus in the apical region. The microscopic study of the epicardial coronary arteries showed atherosclerotic impairment with fibrosis and calcification in atheromatous plaques and important obstruction of the vascular lumen of the major branches (Figure 3). The branches of the left coronary artery showed: maximal luminal obstruction of 75% in the first centimeter of the circumflex artery (CX), and 90% in the fourth centimeter of the anterior interventricular artery (AD). In addition, to aggravate the obstruction of the latter, in the fourth centimeter, there was an old recanalized thrombus in a calcified atherosclerotic plaque. The right coronary artery (RC) showed a maximal obstruction of 60% in its first centimeter, and 70% in the first centimeter of its posterior interventricular branch (PD). Neither recent myocardial infarction nor coronary thrombosis nor intracoronary stent*s* were seen. The lungs, liver and spleen showed morphological changes of chronic passive congestion secondary to CHF (Figure 4). The aorta and cerebral arteries of the Willis polygon evidenced moderate atherosclerosis with calcification. The brain showed neither recent nor old ischemic infarction. There were benign nephrosclerosis, represented by hyalinization of the wall of the glomerular afferent arterioles, and myocardial sclerosis, with multifocal perivascular fibrosis in the left ventricular myocardium, due to systemic arterial hypertension. The lungs showed bilateral suppurative aspiration pneumonia, with particulate food matter and Gram-positive filamentous bacterial aggregates, morphologically compatible with *Actinomyces sp*, which are saprophyte microorganisms of the mouth (Figure 5). There was benign nodular prostatic hyperplasia, accompanied by a distended and thickened urinary bladder, with no morphological evidence of infection. There were acute tubular necrosis in the kidneys, hepatic centrilobular necrosis in the liver, and recent multifocal subendocardial infarctions in both ventricles, resulting from low cardiac output. Although the patient had reported coronary angioplasty with stent implantation 8 years before, no stent was identified in the coronary arteries. Neither malignant neoplasms nor morphological evidence of infection in other organs were found. (Léa Maria Macruz Ferreira Demarchi, MD)

**Anatomopathological diagnoses:** 1) systemic atherosclerosis; 2) coronary atherosclerosis; 3) ischemic heart disease, with healed infarction in the left ventricular anterior and anterolateral walls and in the anterior two-thirds of the ventricular septum; 4) left ventricular aneurysmatic dilatation in the left ventricular anterior wall with an organizing thrombus in the endocardium subjacent to the healed infarction area; 5) congestive heart failure; 6) aspiration pneumonia; 7) mixed hemodynamic shock (cardiogenic/infectious). (Léa Maria Macruz Ferreira Demarchi, MD)

## Comments

Ischemic heart disease (IHD) is the major cause of death in Brazil and worldwide, with higher incidence in men aged 40 years and older.^8,9^ Coronary atherosclerosis is the major contributor to the occurrence of IHD, despite the global preventive measures and the advance in the hemodynamic and pharmacological techniques to treat atherosclerotic disease. Thus, the risk factors for the development of IHD are those for coronary atherosclerosis. This case shows the progression of coronary atherosclerosis and its complications in a male patient with risk factors, such as age (72 years) and systemic arterial hypertension. The atherosclerotic involvement of the coronary arteries, more marked in the AD and CX branches, and the old recanalized thrombus in DA explain the healed transmural infarction in the left ventricular anterior and anterolateral walls and in the ventricular septum, from the heart base to its tip. The complications of myocardial infarction depend on the location and extension of the myocardial necrotic area, which, in our patient, are represented by left ventricular aneurysmal dilation, extensive transmural myocardial fibrosis in the left ventricular anterior wall and organizing thrombus in the endocardium of the infarcted area. An aneurysm can occur early or later after myocardial infarction,^10^ and its presence increases the risk for ventricular arrhythmias and CHF. On postmortem exams, aneurysms are found in cases of extensive myocardial infarction and the hearts are enlarged, with hypertrophy of the remaining left ventricular myocardium, left ventricular dilatation and significant luminal obstruction of the major branches of the epicardial coronary arteries. Congestive heart failure is frequent, being the major cause of death.^11^ The mortality rate of patients of both sexes, aged at least 70 years, who develop CHF after myocardial infarction and have an abnormal left ventricular ejection fraction, varies from 41% to 92%, respectively, from the first to the fifth post-infarction year.^12^ In the case here discussed, the complication cited contributed to CHF, morphologically identified as anasarca, chronic passive congestion of the lungs, liver and spleen, low cardiac output and cardiac cachexia, determining the patient’s unfavorable outcome. The cause of death was hemodynamic shock, to which CHF and aspiration pneumonia contributed. (Léa Maria Macruz Ferreira Demarchi, MD)

**Section editor:** Alfredo José Mansur (ajmansur@incor.usp.br)

**Associated editors:** Desidério Favarato (dclfavarato@incor.usp.br)

Vera Demarchi Aiello (vera.aiello@incor.usp.br)
